# Bioinformatics: The Path to Species Comparison

**DOI:** 10.1289/ehp.112-1277126

**Published:** 2004-08

**Authors:** W. Conard Holton

Systems biology relies on integrating genetic, proteomics, and metabolic data, and on understanding interdependent cellular and intercellular events that are constantly in flux. To accomplish this feat, researchers have relied on DNA and protein sequence databases and high-throughput expression analysis techniques such as microarrays to produce ever-growing libraries of expression data. DNA and protein sequences can be quickly such as BLAST (Basic Local Alignment Search Tool), a program that identifies similar genes in different organisms. Now scientists are applying this computational approach to protein interaction networks, which are the means by which proteins communicate.

“As we move from a focus on sequences to one on networks, we need a tool similar to BLAST,” says Trey Ideker, an assistant professor of bioengineering at the University of California, San Diego. The software program PathBLAST was developed to fill this need by a group consisting of researchers from Ideker’s lab and the lab of Brent Stockwell, now an assistant professor of biological sciences at Columbia University. At the time, both Ideker and Stockwell were fellows at the Whitehead Institute for Biomedical Research in Cambridge, Massachusetts, and worked on the program development with Richard Karp, a professor of bioengineering and mathematics at the University of California, Berkeley, known for his work in combinatorial algorithms and bioinformatics.

The PathBLAST program rapidly compares protein interaction networks across two different organisms using fast-executing algorithms. The program searches for high-scoring alignments involving one path from each network. The proteins of the first path are paired with putative homologs—or proteins presumed to have a common origin and function—from the other species and occurring in the same order in the second path. PathBLAST is built as a plug-in to Cytoscape, a widely used software platform. Scientists use Cytoscape to visualize molecular interaction networks and integrate these interactions with gene expression profiles and other data.

“The important stuff in biology is revealed by comparing things,” says Ideker. “By comparing protein interaction networks of two different species or even within species, we can identify pathways and complexes that have been conserved over evolution.” These evolutionarily conserved pathways allow interpretation of the network of a poorly understood organism based on its similarity to that of a well-known species. This comparison could provide a model of signaling and regulatory pathways that are related to a response to an environmental toxicant. It could also help target drugs to pathways that are present in a pathogenic organism but absent from its human host. Such a model could furthermore help identify drugs that would repair damaged pathways or even cause new ones to be formed.

The PathBLAST development group published a paper in the 30 September 2003 issue of *Proceedings of the National Academy of Sciences* in which they identified the conserved pathways within the yeast *Saccharomyces cerevisiae* and the bacterium *Helicobacter pylori*. For example, the authors found that one pathway that was critical in catalyzing DNA replication and another in protein degradation were conserved in both organisms as a single network. Within seconds, the program had determined that the bacterium contained 1,465 interactions among 732 proteins, and the yeast contained 14,489 interactions among 4,688 proteins.

This report proved that the method works for matching conserved networks from among all the networks in two species, according to software engineer Brian Kelley, a member of Stockwell’s lab. Kelley says, “The next step is to prove the software in a novel application where you start with a given disease network and see if it is conserved in other species. Once you prove this utility, then the use of PathBLAST will skyrocket.” Kelley adds that research into the mTOR cell growth–triggering protein pathway may prove to be that application. This pathway is composed of a complex of proteins that respond to nutrient cues; understanding it will clarify the role that nutrients and metabolism play in disease.

Other researchers have taken a complementary approach by comparing what’s known about a disease to a known network. At Beyond Genomics in Waltham, Massachusetts, researchers measure quantitative differences between transcripts, proteins, and metabolites across a given disease model, determine correlations within the data set, and then compare the experimentally derived network with a known biological network or pathway.

“As the protein interaction databases become more heavily populated with interactions among higher eukaryotes, PathBLAST and related approaches will start to shine as they can help elucidate the set of core biological networks for a given genome,” says Tom Plasterer, the principal scientist for bioinformatics at Beyond Genomics. “These networks—when coupled with a tightly defined experimental context—will be invaluable in understanding mechanisms of disease, where one expects compensatory and subtly differing biological networks to emerge.”

The PathBLAST website is hosted by the Whitehead Institute and available at **http://www.pathblast.org/**; it will soon be mirrored at the San Diego Supercomputer Center at the University of California, San Diego. And as for whether industry will embrace PathBLAST, Ideker says, “It’s still early. Speculating too far about these technologies is like asking industry in 1980, ‘Is genome sequencing going to revolutionize your drug discovery pipeline?’ Even in 2004, the verdict is still out on that one!”

## Figures and Tables

**Figure:**
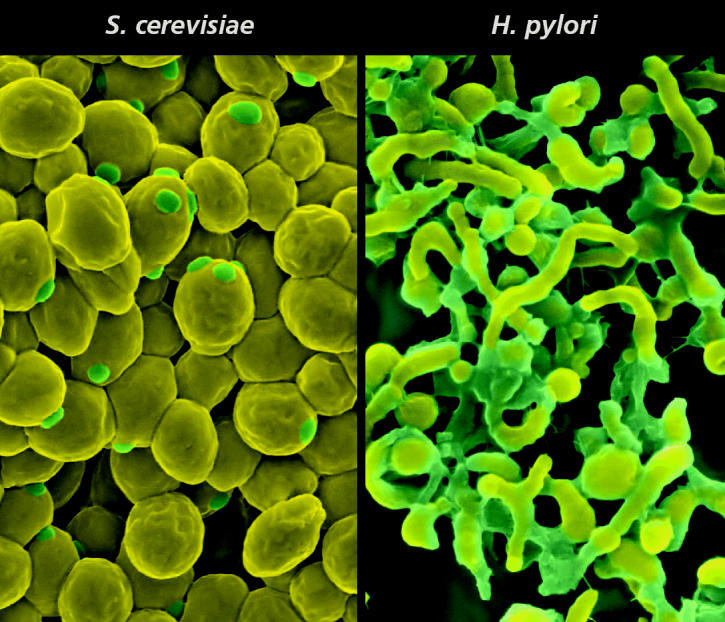
**Conservation comparison.** PathBLAST software allows researchers to identify protein interaction networks that are conserved across multiple species.

